# Impact of the combined loss of BOK, BAX and BAK on the hematopoietic system is slightly more severe than compound loss of BAX and BAK

**DOI:** 10.1038/cddis.2015.304

**Published:** 2015-10-22

**Authors:** F Ke, S Grabow, G L Kelly, A Lin, L A O'Reilly, A Strasser

**Affiliations:** 1 The Walter and Eliza Hall Institute of Medical Research, Melbourne, Victoria, Australia; 2 Department of Medical Biology, The University of Melbourne, Melbourne, Victoria, Australia

## Abstract

It is well established that BAX and BAK play crucial, overlapping roles in the intrinsic pathway of apoptosis. Gene targeted mice lacking both BAX and BAK have previously been generated, but the majority of these animals died perinatally. BOK is a poorly studied relative of BAX and BAK that shares extensive amino acid sequence homology to both proteins, but its function remains largely unclear to date. To determine whether BOK plays an overlapping role with BAX and BAK, we utilized a hematopoietic reconstitution model where lethally irradiated wild type mice were transplanted with *Bok*^*−/−*^*Bax*^*−/−*^*Bak*^*−/−*^ triple knockout (TKO) fetal liver cells, and compared alongside mice reconstituted with a *Bax*^*−/−*^*Bak*^*−/−*^ double knockout (DKO) hematopoietic compartment. We report here that mice with a TKO and DKO hematopoietic system died at a similar rate and much earlier than control animals, mostly due to severe autoimmune pathology. Both TKO and DKO reconstituted mice also had altered frequencies of various leukocyte subsets in the thymus, bone marrow and spleen, displayed leukocyte infiltrates and autoimmune pathology in multiple tissues, as well as elevated levels of anti-nuclear autoantibodies. Interestingly, the additional deletion of BOK (on top of BAX and BAK loss) led to a further increase in peripheral blood lymphocytes, as well as enhanced lymphoid infiltration in some organs. These findings suggest that BOK may have some functions that are redundant with BAX and BAK in the hematopoietic system.

Apoptosis is a highly conserved process for killing unwanted and dangerous cells in multicellular organisms that is critical for several physiological processes, including embryonic development, tissue homeostasis and termination of immune responses.^1, 2, 3^ Defects in apoptosis can contribute to the development of several diseases, including cancer and autoimmune pathologies due to the inappropriate survival of defective cells, or ischemic injury and immunodeficiency caused by excessive cell death.^4, 5^

In mammals, cell death can proceed via the ‘intrinsic' (also called ‘BCL-2 regulated', ‘mitochondrial' or ‘stress') or the ‘extrinsic' (also called ‘death receptor') pathway, both converging upon the activation of caspases that mediate cell demolition.^2, 3, 6, 7, 8, 9^ Activation of the extrinsic pathway is triggered by ligation of ‘death receptors', members of the tumor necrosis factor receptor (TNFR) family with an intra-cellular ‘death domain' on the surface of the cell.^2^ Conversely, the intrinsic pathway is triggered by developmental cues, growth factor or nutrient deprivation, and a broad range of cytotoxic stimuli. This pathway is regulated by the BCL-2 family of proteins.^10^

Members of the BCL-2 family can be classified into three subgroups: the pro-survival proteins (BCL-2, MCL-1, BCL-X_L_, BCL-W, A1) that are critical for cell survival; the BAX/BAK-like multi-BH domain containing pro-apoptotic proteins (BAX, BAK, BOK) that permeabilize the mitochondrial outer membrane (MOMP), and the pro-apoptotic BH3-only proteins (BIM, BID, PUMA, NOXA, BIK, BAD, BMF, HRK) that initiate signaling through the intrinsic apoptotic pathway.^3, 10, 11^ BH3-only proteins are transcriptionally and/or post-translationally activated upon exposure of cells to a cytotoxic insult (e.g., cytokine deprivation, *γ*-irradiation). They can activate BAX/BAK either by binding to them directly, or indirectly by binding to pro-survival BCL-2 members, thus liberating BAX and BAK to permeabilize the MOMP.^12, 13^ This constitutes the ‘point-of-no-return' in the intrinsic apoptotic pathway, leading to the release of apoptogenic molecules, such as cytochrome C and Smac/Diablo, which promote activation of the caspase cascade to cause cell death.^14^

Much of what is known about the crucial and largely overlapping roles of BAX and BAK in apoptosis (particularly in the hematopoietic system) has been learnt from studies of gene-targeted mice that lack these proteins. Mice lacking BAK alone are largely normal, with the exception of a slight increase in platelets due to its role in determining the lifespan of these anuclear cell fragments.^15^ BAX-deficient mice display mild lymphoid hyperplasia, a minor increase in neurons in certain parts of the brain, and male sterility, but otherwise appear normal.^16, 17, 18^ These findings indicate that BAX and BAK are mostly functionally redundant. Indeed, more than 90% of *Bax*^*−/−*^*Bak*^*−/−*^ compound mutant mice die perinatally for still unknown reasons. The few survivors exhibited several phenotypic abnormalities, including webbed feet, substantial neuronal and lymphoid cell accumulation, as well as profound resistance of their lymphocytes (and other cell types) to a broad range of apoptotic stimuli.^17, 19, 20^ Perhaps surprisingly, several tissues in these *Bax*^*−/−*^*Bak*^*−/−*^ mutants such as the bladder, liver, heart and pancreas still developed normally and functioned at physiological levels,^17^ suggesting that another protein may be involved in the activation of the effector phase of apoptosis in some circumstances. One such candidate is BOK, a BCL-2 family member showing >70% amino acid sequence homology to BAX and BAK ^21, 22^

BOK was first isolated from a yeast two-hybrid screen as a protein that interacted with MCL-1.^21^ Subsequent *in vitro* studies demonstrated that enforced BOK overexpression could induce apoptosis, suggesting that it functions in a pro-apoptotic manner, similar to BAX and BAK. Our laboratory has generated *Bok*^*−/−*^ mice and found that these animals are healthy, fertile and normal in appearance.^22^ Even when BOK was deleted in combination with BAX (*Bok*^*−/−*^*Bax*^*−/−*^) or BAK (*Bok*^*−/−*^*Bak*^*−/−*^), only minor effects were observed, most notably the increased persistence of primordial follicle oocytes in aged *Bok*^*−/−*^*Bax*^*−/−*^ females. Taken together, these findings imply that BOK may act in a manner that is fully redundant with the functions of BAX and BAK, or may have a function that is yet unknown.

Due to the difficulty in generating BOK/BAX/BAK triple knockout mice, we examined the consequences of combined loss of BOK, BAX and BAK in hematopoietic cells using a reconstitution model. This allowed us to examine a sufficient number of animals, as well as assess the long-term physiological consequences of harboring hematopoietic cells deficient for all three of these multi-BH domain pro-apoptotic proteins.

We report that chimeric mice with a *Bok*^*−/−*^*Bax*^*−/−*^*Bak*^*−/−*^ TKO hematopoietic system, similar to *Bax*^*−/−*^*Bak*^*−/−*^ DKO hematopoietic chimeras, developed splenomegaly with a significant increase in blood lymphocytes, as well as autoantibodies against nuclear antigens and multiple tissues, leading to severe SLE-like autoimmune disease. Our findings suggest that BOK plays a minor role in the hematopoietic system that overlaps with BAX and BAK.

## Results

### *Bok*^−/−^*Bax*^−/−^*Bak*^−/−^ TKO hematopoietic chimeras die at a similar rate as *Bax*^−/−^*Bak*^−/−^ DKO chimeras

**Figure 1:**
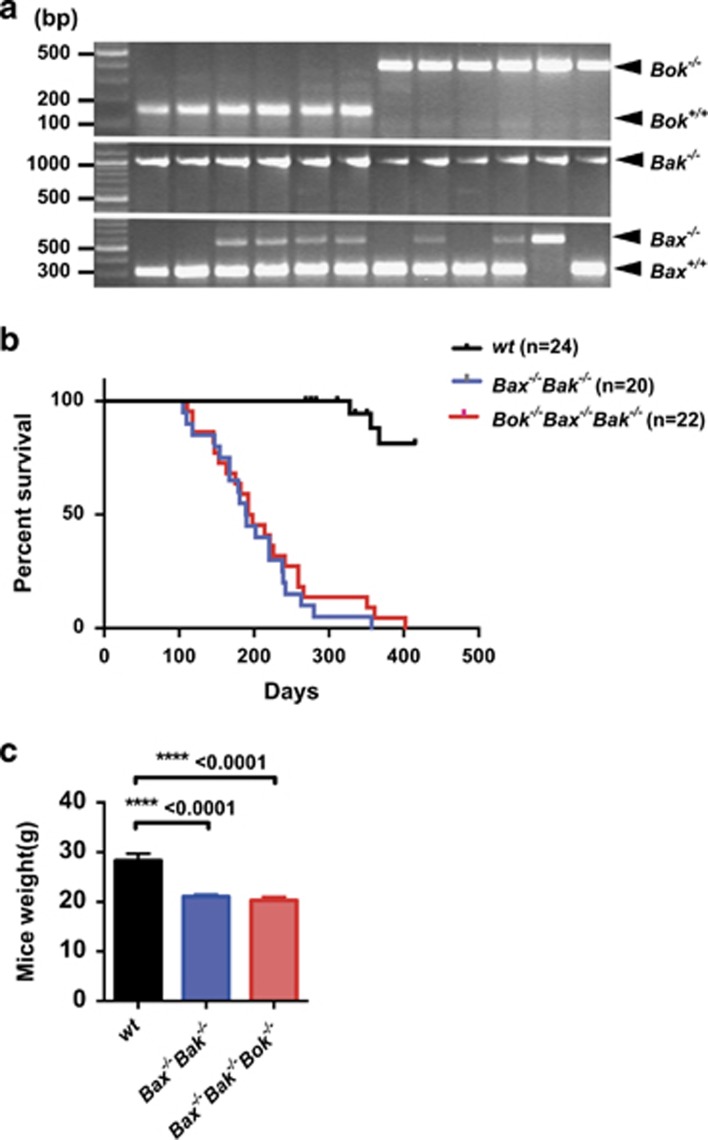
Mice reconstituted with *Bok*^*−/−*^*Bax*^*−/−*^*Bak*^*−/−*^(TKO) fetal liver cells die abnormally early. (**a**) Embryos were harvested from *Bax*^*+/*^*^−^Bak*^*−/−*^ and *Bok*^*−/−*^*Bax*^*+/*^*^−^Bak*^*−/−*^intercrosses and genotyped to identify *Bax*^*−/−*^*Bak*^*−/−*^ and *Bok*^*−/−*^*Bax*^*−/−*^*Bak*^*−/−*^offspring, respectively. The deleted or wt *Bok* alleles generate bands at 383 base pairs (bp) and 150 bp, respectively. The absence of *Bak* produces a 1000 bp product, while the deleted or wt alleles of *Bax* are detected at 507 bp and 304 bp, respectively, by gel electrophoresis. (**b**) Kaplan–Mayer survival curves for wt (*n*=24, TKO (*n*=22) and DKO (*n*=20) chimeric mice. Wt control *versus Bax*^*−/−*^*Bak*^*−/−*^ DKO: *P*<0.0001; wt control *versus Bok*^*−/−*^*Bax*^*−/−*^*Bak*^*−/−*^TKO: *P*<0.0001. (**c**) TKO and DKO chimeras exhibited significant weight loss at the time of sacrifice. Significant differences (i.e. *P*<0.05) are indicated by *P*-values above brackets

Due to the difficulty in obtaining TKO mice after an extended period of breeding, timed matings of *Bok*^*−/−*^*Bax*^*+/*^*^−^Bak*^*−/−*^ and *Bax*^*+/*^*^−^Bak*^*−/−*^ intercrosses on a C57BL/6-Ly5.2 background were established to obtain E14 *Bok*^*−/−*^*Bax*^*−/−*^*Bak*^*−/−*^ (TKO) and *Bax*^*−/−*^*Bak*^*−/−*^ (DKO) embryos respectively. Following genotyping (Figure 1a), fetal liver cells (FLCs) containing hematopoietic stem cells were extracted from TKO and DKO embryos and injected into lethally irradiated C57/BL6-Ly5.1 wild-type recipients. For comparison, a cohort of C57BL/6-Ly5.1 recipients was injected with wild-type (C57BL/6-Ly5.2) FLCs (hereon referred to as control mice). Reconstituted animals were monitored daily for signs of illness including hunching, blood in urine, labored breathing and enlarged lymphoid organs. To ensure that these recipient mice (also called chimeras, or chimeric mice in our study) had been successfully reconstituted with donor hematopoietic precursor cells, flow cytometric analysis was performed on their lymphoid organs at 9–12 weeks post-transplantation to determine the percentage of leukocytes expressing the Ly5.2 marker. In all recipients, more than 90% of leukocytes in the spleen, thymus and lymph nodes were Ly5.2+. We also observed no differences in reconstitution efficiency regardless of whether donor cells were derived from WT, DKO or TKO embryos.

The control mice that were injected with WT FLCs remained healthy throughout the study (415 days post reconstitution, *n*=24), and were examined alongside sick TKO and DKO chimeric animals. In contrast, we observed a substantially reduced lifespan in mice reconstituted with either *Bax*^*−/−*^*Bak*^*−/−*^ (median survival: 190 days, range: 105–357 days, *n*=20, *P*<0.0001 *versus* WT) *or Bok*^*−/−*^*Bax*^*−/−*^*Bak*^*−/−*^(median survival: 195 days, range: 111–402 days, *n*=22, *P*<0.0001 *versus* WT) FLCs (Figure 1b). These animals were sacrificed when ill, and displayed significant weight loss compared with control mice (Figure 1c). However, no differences in survival or severity of weight loss were observed between sick TKO and DKO chimeric mice.

### Mice reconstituted with *Bok*^−/−^*Bax*^−/−^*Bak*^−/−^ TKO FLCs contain abnormally increased numbers of leukocytes in the periphery

To determine whether the additional loss of BOK on top of the loss of BAX and BAK affected leukocyte homeostasis in the blood, heart bleeds were obtained from sick TKO and DKO chimeric mice for analysis, or at termination of the experiment for control animals.

**Figure 2:**
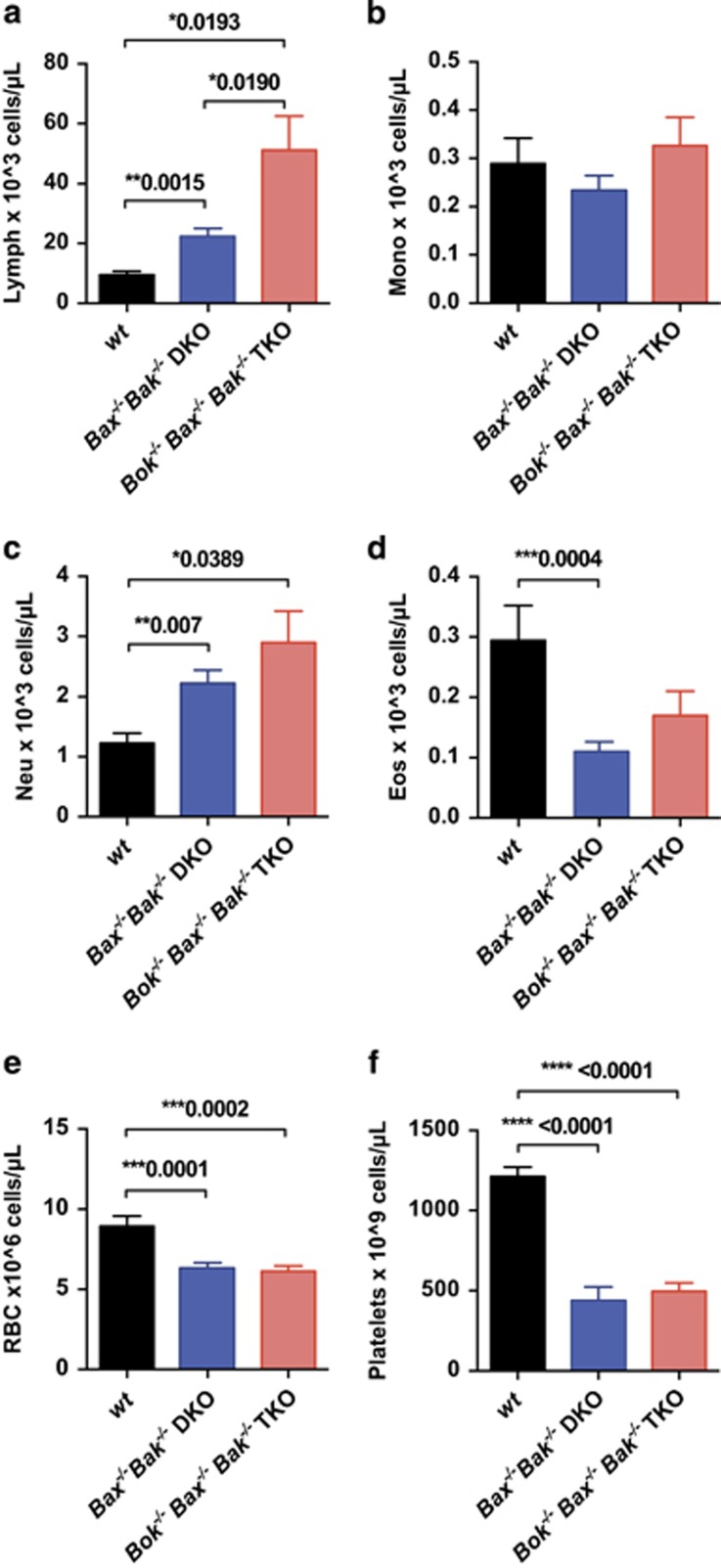
The compound loss of BOK/BAX/BAK in the hematopoietic compartment leads to an increase in peripheral blood lymphocytes. Blood analysis from sick TKO (*n*=19) and DKO (*n*=19) chimeras and healthy wt controls (*n*=9) was performed using the ADVIA automated machine to determine the numbers of (**a**) lymphocytes, (**b**) monocytes, (**c**) neutrophils, (**d**) eosinophils, (**e**) red blood cells and (**f**) platelets. Bar graphs depict the mean±S.E.M. of the total numbers of cells of a particular subset. *P*-values above brackets indicate significant differences (i.e., *P*<0.05)

Consistent with earlier findings,^23^ we observed that DKO chimeric animals had approximately twice the numbers of peripheral blood lymphocytes (Figure 2a) and decreased platelet counts (Figure 2f) compared with mice reconstituted with WT FLCs. The TKO chimeric animals, like those reconstituted with DKO FLCs, also displayed mild anemia (Figure 2e) and thrombocytopenia (Figure 2f), elevated neutrophils (Figure 2c), but no significant increases in monocytes or eosinophils compared with the control WT chimeras (Figures 2b–d). Interestingly, triple deficiency for BOK/BAX/BAK in the hematopoietic compartment caused an even more pronounced increase in lymphocyte numbers compared with the compound loss of BAX/BAK alone (Figure 2a). These results imply that BOK may have a minor role in the maintenance of lymphocyte homeostasis.

### Chimeric mice harboring a TKO or DKO hematopoietic system have abnormally increased numbers of various leukocyte subsets in the spleen, thymus and bone marrow

Since *Bok*^*−/−*^*Bax*^*−/−*^*Bak*^*−/−*^ TKO reconstituted mice displayed a significant increase in blood lymphocytes, we next examined whether they also had abnormally increased numbers of leukocyte subsets in their primary and secondary lymphoid organs. We therefore performed cell counting and FACS analysis of the spleen, thymus and bone marrow of sick chimeric mice, and compared them alongside healthy age-matched WT chimeric controls.

**Figure 3:**
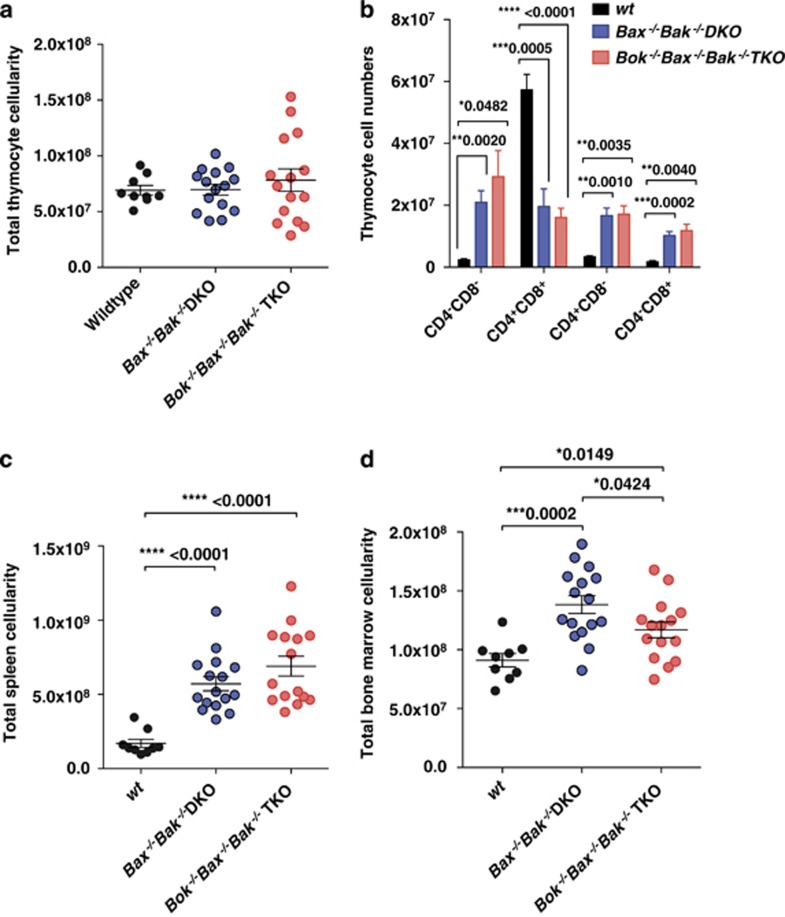

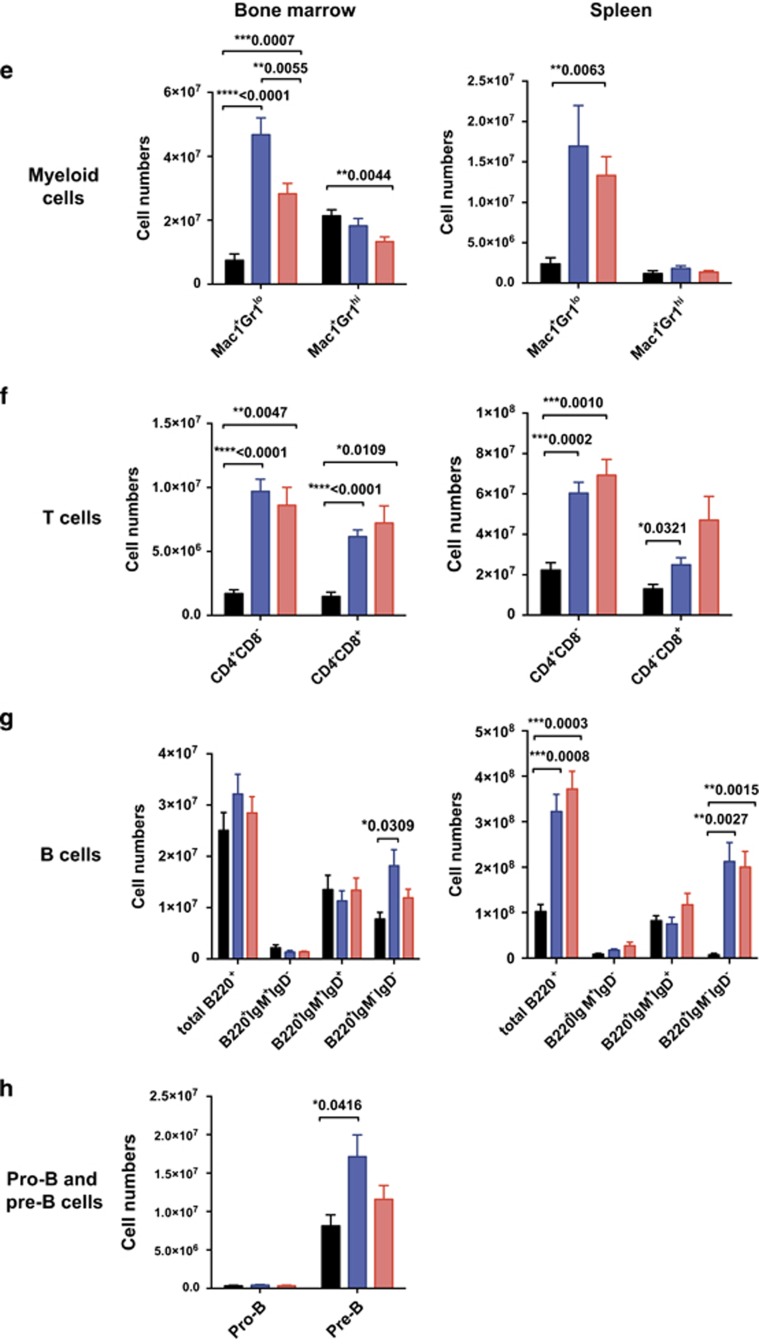
TKO and DKO hematopoietic chimeric mice have increased numbers of select leukocyte subsets in the thymus, spleen and bone marrow. (**a**) Total thymic, (**c**) splenic and (**d**) bone marrow cellularity of wt (*n*=8), *Bax*^*−/−*^*Bak*^*−/−*^ DKO (*n*=15), and *Bok*^*−/−*^*Bax*^*−/−*^*Bak*^*−/−*^TKO (*n*=15) FLC reconstituted mice. (**b**) Thymic subsets of sick DKO (*n*=10) and TKO (*n*=13) chimeras were determined by flow cytometric analysis alongside similarly aged healthy wt controls (*n*=6). (**e**–**h**) The numbers of leukocyte populations (Mac1^+^Gr1^lo^ macrophages, Mac1^+^Gr1^hi^ granulocytes, CD4^+^CD8^−^ and CD4^−^CD8^+^ mature T-cells, B220^+^ sIgM^hi^ sIgD^lo^ and B220^+^ sIgM^lo^ sIgD^hi^ mature B, B220^+^ sIgM^-^ sIgD^-^ memory B, B220^+^sIg^−^ c-Kit^−^ pre-B and B220^+^sIg^-^ c-Kit^+^ pro-B cells) in the spleen and/or bone marrow of reconstituted mice were examined by cell counting and flow cytometry. Results represent mean±S.E.M. of total cell numbers of a given subset. *P*-values above brackets indicate significant differences (i.e., *P*<0.05)

Total thymic cellularity was comparable between mice reconstituted with TKO, DKO or WT FLCs (Figure 3a). However, the representation of thymic cell subsets from TKO or DKO chimeras were altered, characterized by increased numbers of CD4^−^CD8^−^, CD4^+^CD8^−^, and CD4^−^CD8^+^ T-lymphoid cells, and a decrease in immature CD4^+^CD8^+^ thymocytes compared with control animals (Figure 3b). Interestingly, one mouse reconstituted with DKO FLC developed a thymic lymphoma and was excluded from our dataset due to a dramatic increase in total thymus cellularity, which was mostly accounted for by CD4^lo/^^−^CD8^+^ lymphoma cells.

TKO and DKO chimeras also displayed splenomegaly compared with control mice, and this was reflected by a marked increase in total splenic cellularity (Figure 3c). FACS analysis revealed that this could be attributed to an increase in the numbers of B220^+^ B cells, mature CD4^+^CD8^−^ as well as CD4^−^CD8^+^ T-lymphocytes and macrophages (Mac1^+^Gr1^lo^) (Figures 3e–g). The population of splenic B cells that were most significantly elevated displayed a B220^+^IgM^−^IgD^−^ phenotype, suggesting a marked increase in memory B cells (Figure 3g). This observation is consistent with earlier studies showing that *Bax*^*−/−*^*Bak*^*−/−*^ DKO mice similarly had increased numbers of B220^+^IgD^−^ cells.^17^

Both TKO and DKO chimeras also had increased total bone marrow cellularity compared with control WT chimeras (Figure 3d). Flow cytometric analysis revealed that this could be attributed to an increase in macrophages, CD4^+^ and CD8^+^ mature T lymphocytes, pre-B cells and B220^+^IgM^−^IgD^−^ B cells (which were also detected in the spleen) (Figures 3e–h). Collectively, these results showed that compound loss of BOK, BAX and BAK in the hematopoietic compartment causes a small but significant increase in the accumulation of blood lymphocytes, but similar alterations in leukocyte subset composition in the spleen, thymus and bone marrow compared with the combined absence of BAX and BAK.

### TKO and DKO chimeric mice develop lymphocytic infiltration in multiple organs and organ-specific autoantibodies

**Figure 4:**
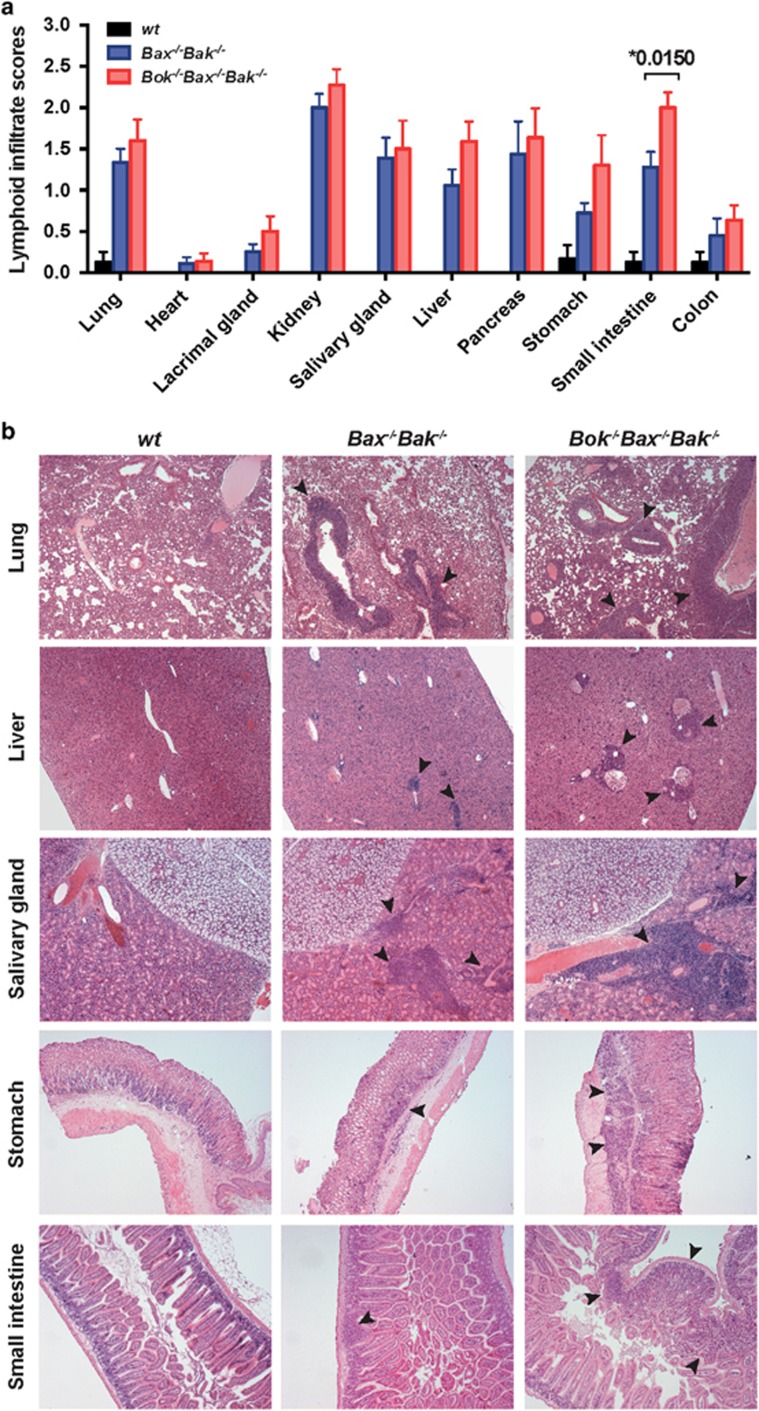
TKO hematopoietic chimeras display substantial lymphocytic infiltration in several organs. (**a**) Summary of the incidence of lymphocytic infiltration (scored on a scale of 0–3) into the indicated organs. Bar graphs represent mean±S.E.M of score for each tissue from mice reconstituted with wt (*n*=4), *Bax*^*−/−*^*Bak*^*−/−*^DKO (*n*=9) or *Bok*^*−/−*^*Bax*^*−/−*^*Bak*^*−/−*^TKO (*n*=11) FLCs, *P*<0.05. Tissues were harvested at the time when mice were sick (for DKO and TKO chimeras) or at termination of the experiment (for wt controls). (**b**) Representative H&E stained sections depicting leukocyte infiltration in the lung, liver, salivary glands, stomach and small intestine of mice reconstituted with FLCs of the indicated genotype. Arrows indicate areas of infiltration (Magnification: × 5)

Earlier studies have demonstrated that mice reconstituted with DKO FLCs developed inflammation and necrotizing vasculitis in several organs.^23^ To assess whether the additional loss of BOK (on top of loss of BAX and BAK) in hematopoietic cells would lead to more severe disease, various tissues from sick TKO chimeras were examined blinded, and scored on a scale of 0–3 for lymphocytic infiltration (tissues from WT control and *Bax*^*−/−*^*Bak*^*−/−*^DKO FLC reconstituted mice were included for comparison). The results showed that although both TKO and DKO chimeric animals exhibited substantial lymphocytic infiltration in multiple organs (Figure 4a), some tissues from TKO chimeras, such as the small intestine, displayed notably increased infiltration compared with the corresponding tissues from DKO chimeras.

**Figure 5:**
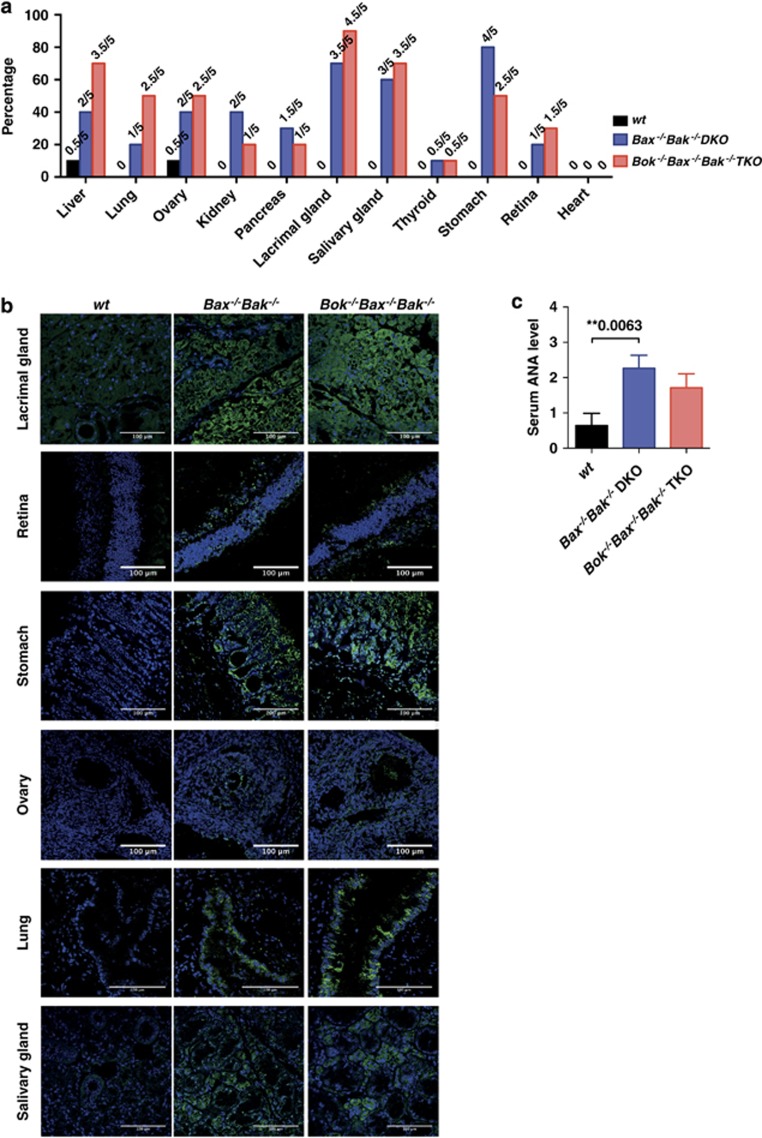
Mice with a TKO or DKO hematopoietic system developed autoimmune disease with autoantibodies against nuclear antigens and several tissues. (**a**) Percentages of mice that harbor autoantibodies against the tissues specified. Numbers above bars represent the tabulated scores for each tissue stained with sera from wt, DKO or TKO chimeric mice (*n*=5 per genotype examined). (**b**) Representative images of immunofluorescent staining of the lacrimal gland, retina, stomach, ovary, lung and salivary gland from *Rag-1*^*−/−*^ mice stained with sera from the chimeric mice indicated. (Scale bars, 100 *μ*m). Nuclei are revealed by DAPI staining (blue), while IgM, IgG and IgA organ-specific autoantibodies are labeled in green. (**c**) The levels of anti-nuclear antibodies in the sera from chimeric mice of the indicated genotypes were determined using an ELISA kit assay. Data represent mean±S.E.M., *n*=8–9 mice per genotype. *P*-values above brackets indicate significant differences (i.e., *P*<0.05)

We next examined whether the lymphocytic tissue infiltration in sick DKO and TKO reconstituted animals was associated with the production of organ-specific autoantibodies. This was investigated by immune-staining a panel of frozen tissue sections from *Rag-1*^*−/−*^mice (to prevent background staining by endogenous immunoglobulins) with sera from WT control, TKO or DKO chimeras, and visualized using confocal microscopy (*n*=5 reconstituted mice per genotype). Tissues that stained strongly, moderately, or not at all were given a score of +1, +0.5 or 0, respectively (Figure 5a). The combined absence of BOK/BAX/BAK in hematopoietic cells caused the production of autoantibodies against many tissues, such as the lacrimal and salivary glands, liver, stomach, lung and ovary (Figure 5b). This autoimmune pathology was also observed in BAX/BAK DKO reconstituted mice, but not in similarly aged WT chimeras (Figure 5b).

We also tested for the presence of anti-nuclear autoantibodies (ANA) in sera from sick DKO and TKO chimeric mice. To ensure adequate comparison, we selected animals that had survived for a similar period of time post hematopoietic reconstitution before they had to be taken due to illness. Control animals were also analyzed at a comparable age or even older, but did not exhibit any signs of disease. Results revealed increased ANA levels in sick DKO and TKO chimeras compared with healthy WT controls, indicating the presence of elevated autoantibodies to chromatin (dsDNA and histones) (Figure 5c).

### BOK/BAX/BAK-deficient leukocytes are resistant to diverse apoptotic stimuli

Previous studies have shown that the absence of BAX,^16^ BAK^17^ or BOK ^22^ alone, as well as the compound loss of BOK/BAX and BOK/BAK ^24^ did not have substantial impact on the response of hematopoietic cells to apoptotic stimuli. However, *Bax*^*−/−*^*Bak*^*−/−*^DKO lymphocytes (and other cell types) are remarkably resistant to a wide variety of cytotoxic stimuli because of a defect in apoptosis.^17^

**Figure 6:**
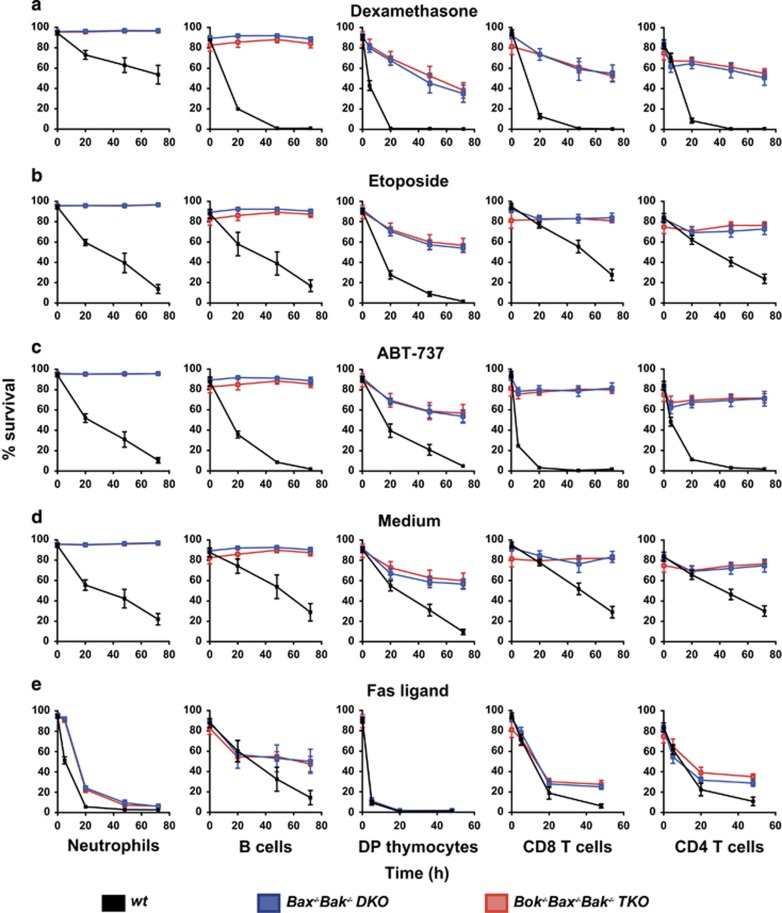
Responses of TKO and DKO lymphocytes to apoptotic stimuli in culture. Mac1^+^Gr1^hi^ neutrophils, B220^+^ B cells, CD4^+^CD8^-^ and CD4^-^CD8^+^ mature T-cells as well as CD4^+^CD8^+^ double positive (DP) immature thymocytes were FACS sorted from the lymph nodes, thymus and bone marrow of wt, DKO and TKO chimeras. These leukocytes were exposed in tissue culture to a wide range of apoptotic stimuli, including (**a**) dexamethasone (1 *μ*M), (**b**) etoposide (1 *μ*M), (**c**) the BH3 mimetic ABT-737 (240 nM), (**d**) growth factor deprivation (DMEM+10% FCS without the addition of cytokines), or (**e**) crossed-linked FasL (100 ng/ml). Cell survival was assayed at the indicated time points by staining with PI and Annexin-V, followed by flow cytometric analysis

To test whether the additional deletion of BOK on top of the loss of BAX and BAK further increased resistance to apoptotic stimuli, we isolated immature double positive (DP) thymocytes (CD4^+^CD8^+^), CD4^+^CD8^-^ and CD4^-^CD8^+^ mature T-cells and B220^+^ B cells from the lymph nodes, as well as bone marrow-derived neutrophils (Mac1^+^Gr1^hi^) from WT control, DKO and TKO chimeric animals, and exposed them to a range of cytotoxic stimuli (Figures 6a–e). We found that although the combined deficiency of BOK/BAX/BAK in lymphoid and myeloid cells conferred a substantial survival advantage over WT control cells, there were no differences in the rate of cell death between *Bok*^*−/−*^*Bax*^*−/−*^*Bak*^*−/−*^ TKO and *Bax*^*−/−*^*Bak*^*−/−*^DKO leukocytes. These results demonstrate that BAX and BAK are the major activators of the effector phase of apoptosis in lymphoid and myeloid cells, and that BOK has only a minor role.

## Discussion

Since its discovery almost two decades ago, BOK has remained a poorly studied member of the BCL-2 family.^21^ Exhibiting a high degree of amino acid sequence homology to BAX and BAK, BOK was postulated to perform a similar pro-apoptotic role in the intrinsic apoptotic pathway. Indeed, several *in vitro* studies, using a wide variety of cell lines, such as Chinese hamster ovary (CHO) cells,^21^ primary neurons,^25^ mouse embryonic fibroblasts (MEFs),^26^ HeLa cells (Zhang *et al.*, 2009), HC11 mammary epithelial cells^27^ and HEK293T cells^25, 28^ demonstrated that BOK overexpression could induce apoptosis.

However, *Bok*^*−/−*^ animals appear normal, and their hematopoietic cells have no survival advantage over WT controls when challenged with a range of apoptosis inducing stressors.^22^ This does not exclude a pro-apoptotic role for BOK, since many studies have similarly shown that overexpressed BAK or BAX can induce apoptosis, but singly deficient *Bak*^*−/−*^ and *Bax*^*−/−*^ mice showed only minor developmental abnormalities, and their cells were largely normally sensitive to apoptotic stimuli.^16, 17^ These studies emphasized the importance of examining the overlap in functions amongst the multi-BH domain pro-apoptotic BCL-2 family members.

The dramatic developmental abnormalities observed in *Bax*^*−/−*^*Bak*^*−/−*^ DKO mice highlight the functional redundancy between BAX and BAK.^17^ Nonetheless, the survival of a small number of DKO animals to adulthood and the normal morphogenesis of multiple tissues in these survivors (that are assumed to be shaped by apoptosis) implied that related proteins, or BAX/BAK-independent mechanisms exist for cell death to proceed. To test whether BOK may have roles that overlap with those of BAX and BAK, we established matings to generate *Bok*^*−/−*^*Bax*^*−/−*^*Bak*^*−/−*^(TKO) offspring. However, since no TKO pups were obtained after several months of breeding, we utilized a hematopoietic reconstitution model instead to determine the consequences of BOK/BAX/BAK loss in a physiological context. This method had previously been employed to determine the impact of combined loss of BAX and BAK in hematopoietic cells.^23^ Additionally, the functions of many other pro-apoptotic as well as pro-survival BCL-2 family members had initially been revealed through examination of the hematopoietic compartment of gene-targeted mice.^29, 30, 31, 32, 33^

Our findings revealed that both TKO and DKO chimeras had a similarly shortened lifespan compared with control (animals reconstituted with WT FLCs) mice. This indicates that the removal of BOK on top of BAX and BAK in the hematopoietic compartment does not further exacerbate the autoimmune pathologies that are responsible for the demise of BAX/BAK DKO chimeras.^23^ A detailed flow cytometric analysis of the thymus, spleen and bone marrow from DKO and TKO chimeric mice revealed comparable alterations in particular leukocyte populations, such as elevated numbers of macrophages, mature T-cells (both CD4^+^CD8^−^ and CD4^−^CD8^+^), and B cells that exhibited a memory cell phenotype. In the thymus, we noted that both DKO and TKO chimeras showed a significant decrease in double positive (DP) thymocytes (CD4^+^CD8^+^), but a substantial increase in single positive (CD4^+^CD8^−^ and CD4^−^CD8^+^) and double negative (DN) thymocytes (CD4^−^CD8^−^) in comparison to WT controls. Earlier reports have shown that the combined absence of BAX and BAK causes a significant reduction in the proliferation of immature DN progenitors in the thymus^34^ (possibly as a consequence of the over-abundance of mature single positive thymocytes that may compete for growth factors and nutrients), leading to the observed decrease in double positive thymocytes. The increase in single positive *Bax*^*−/−*^*Bak*^*−/−*^ thymocytes can be explained by the enhanced survival of these cells (for instance when deprived of growth factors), as well as their escape from apoptosis induced by negative selection, and/or failure of positive selection.^23, 34^ However, the additional loss of BOK on top of BAX and BAK deletion does not exacerbate the abovementioned thymic abnormalities, suggesting that BOK does not play a major role in the control of thymocyte death.

Interestingly, we found that TKO chimeras exhibited a significant elevation in peripheral blood lymphocytes compared to the DKO chimeras, which is likely attributed to the additional loss of a pro-apoptotic role of BOK. This lymphocytosis may also account for the enhanced lymphoid infiltration observed in several tissues of TKO chimeric mice compared with DKO chimeric animals. We postulate that the circulating lymphocytic population would consist of both B and T-cells, since sick DKO and TKO chimeric animals displayed a pronounced increase in B220^+^ B cells, as well as mature CD4^+^ and CD8^+^ T-cells in the spleen as revealed by flow cytometry. This is most likely due to the failure of lymphocytes to be eliminated following an immune response, therefore leading to their increase in the blood. Consistent with this, several studies examining the compound loss of BAX and BAK in the hematopoietic system similarly showed an accumulation of B and T-cells that displayed a memory phenotype due to their protection from apoptosis.^17, 23, 34^

What could possibly explain the increased blood lymphocytes observed in TKO chimeras compared with DKO chimeras? Earlier studies have shown that lymphocytes are under more stress when traveling through the blood or when they infiltrate organs, because they are removed from survival promoting factors, such as IL-7 and BAFF, produced by stromal cells in the primary and secondary lymphoid tissues. Therefore, losing an additional pro-apoptotic protein (i.e., BOK) in addition to losing BAX and BAK would confer a more prominent survival advantage over *Bax*^*−/−*^*Bak*^*−/−*^ DKO lymphocytes outside of the lymphoid organs. Of note, cell survival assays in tissue culture, which demonstrated similar resistance of TKO and DKO leukocytes to a broad range of cytotoxic stimuli were conducted using cells from lymphoid organs.

These results taken together suggest that in the hematopoietic system, BOK plays a minor role that overlaps with BAX/BAK. Previous studies have shown that BOK is ubiquitously expressed in murine organs, but most abundant in the brain, reproductive tissues and intestine. Hence, it is possible that the function of BOK is more pronounced in certain tissues. In this regard, marked differences in the response to apoptotic stimuli between TKO and DKO cells may only be seen in particular cell types. For instance, aged *Bok*^*−/−*^*Bax*^*−/−*^ females were found to retain increased numbers of ovarian primordial follicles that failed to undergo atresia compared with *Bax*^*−/−*^, *Bok*^*−/−*^ and WT controls, supporting the notion that BOK may play a role in the reproductive tissues.^24^

We also still cannot exclude the possibility that BOK may have different functions to BAX and/or BAK. Prior work using MEFs and growth factor-dependent myeloid progenitors demonstrated that cell killing induced by BOK overexpression could not occur in the absence of BAX or BAK,^26^ suggesting that BOK may behave more like a BH3-only protein that acts upstream of BAX/BAK. Earlier studies with H1299 lung cancer cell lines similarly suggested that BOK functions as a trigger that sensitizes cells to undergo apoptosis upon drug treatment, rather than act as an effector of cell death.^35^ Intriguingly, certain cell types from *Bok*^*−/−*^ mice were shown to die at a faster rate compared with WT controls when treated with Brefeldin A, a drug that induces Golgi/ER stress. In conclusion, our findings and those of others still imply that BOK may exert multiple functions, some that overlap with the pro-apoptotic role of BAX/BAK, and others that remain to be determined.

## Materials and Methods

### Mice

All animal experiments were performed with approval from the Walter and Eliza Hall Institute Animal Ethics Committee, and according to the Australian code of practice for the care and use of animals for scientific purposes.

Singly deficient *Bok*^*−/−*^, *Bax*^*−/−*^ and *Bak*^*−/−*^ mice have previously been described.^16, 17, 22^ The *Bax*^*−/−*^ and *Bak*^*−/−*^ mice were originally generated on a mixed C57BL/6x129SV background using 129SV derived ES cells but have been backcrossed to C57BL/6 mice for >10 generations. The *Bok*^*−/−*^ mice were generated on an inbred C57BL/6 background using C57BL/6 derived ES cells.^22^ *Bax*^*+/−*^*Bak*^*−/−*^ and *Bok*^*−/−*^*Bax*^*+/−*^*Bak*^*−/−*^animals were produced by intercrossing *Bok*^*−/−*^, *Bax*^*+/−*^ and *Bak*^*−/−*^ mutants.

### Hematopoietic reconstitution and blood analysis

Wild-type C57BL/6-Ly5.1 recipient mice were lethally irradiated to deplete the hematopoietic compartment by exposure to 2 × 5.5 Gy *γ*-radiation from a ^60^Co source, 3 h apart.

*Bax*^*+/-*^*Bak*^*−/−*^ intercrosses, as well as *Bok*^*−/−*^*Bax*^*+/-*^*Bak*^*−/−*^ intercrosses were set up to obtain embryonic day 14 (E14) *Bax*^*−/−*^*Bak*^*−/−*^ (DKO) and *Bok*^*−/−*^*Bax*^*−/−*^*Bak*^*−/−*^ (TKO) embryos, respectively. Genotyping analyses to identify *Bax*^*−/−*^*Bak*^*−/−*^ DKO and *Bok*^*−/−*^*Bax*^*−/−*^*Bak*^*−/−*^ TKO embryos were performed using a three-primer PCR reaction as previously described.^22^ All gene-targeted mice/embryos carry the Ly5.2 congenic marker. Fetal livers were extracted from embryos of the appropriate genotypes and single cell suspensions prepared in 600 *μ*l of PBS. This suspension was injected into the tail vein of three C57BL/6-Ly5.1 recipient mice at 2 h post *γ*-irradiation (approximately 3–4 × 10^6^ cells were injected per recipient).

Reconstitution of the hematopoietic system was allowed to take place for at least 8 weeks before mice were used for experiments. Reconstituted mice were closely monitored daily for signs of ill health, and were deemed sick when they presented with features including weight loss, hunched posture, enlarged spleen, labored breathing and/or blood in urine.

Peripheral blood was collected by cardiac puncture after anesthesia, and analyzed with the ADVIA automated hematology system (Bayer, Leverkusen, Germany).

### Immunofluorescence staining and flow cytometric analysis

Single cell suspensions of the thymus, spleen and bone marrow were prepared and enumerated using an automated CASY counter (Scharfe, Krämerstraβe, Reutlinger, Germany). The cellular compositions of these lymphoid organs were determined by staining cells with fluorochrome (R-PE, APC or FITC)-conjugated monoclonal antibodies against cell subset specific surface markers that included anti-CD4 (YTA321 or H129), anti-CD8 (YTS169), anti-B220 (RA3-6B2), anti-IgM (5.1), anti-IgD (1126C), anti-c-Kit (ACK-4), anti-Mac-1 (MI/70), anti-Gr-1 (RB6-8C5) and anti-Ter119 (Ter119). Stained cells were subjected to flow cytometric analysis using an LSR1 machine (Becton Dickinson, Franklin Lakes, NJ, USA).

### Immunofluorescent staining of tissue sections and confocal microscopy

Detection of immune complexes by immunofluorescent staining has previously been described.^23^ Briefly, 5 *μ*m frozen sections of the liver, lung, ovary, kidney, eye, stomach, heart, thyroid, pancreas, lacrimal glands and salivary glands were obtained from *Rag-1*^*−/−*^ mice (to prevent background staining produced by endogenous immunoglobulins), and incubated with sera (1/100 dilution) from sick *Bax*^*−/−*^*Bak*^*−/−*^ and *Bok*^*−/−*^*Bax*^*−/−*^*Bak*^*−/−*^ hematopoietic chimeras. Bound autoantibodies were revealed by secondary staining with FITC-conjugated goat antibodies specific to mouse IgG, IgA and IgM (Cappel, MP Biochemicals, Santa Ana, CA, USA). Staining with DAPI (Sigma, St. Louis, MO, USA) was used to reveal nuclei, and slides were examined using a Leica SP8 confocal microscope (Leica, Wetzlar, Hesse, Germany).

### Analysis of serum ANA levels

Sera obtained from sick DKO and TKO chimeric mice and similarly aged healthy WT chimeras were diluted 1/200 in water, and incubated with the QUANTA Lite ANA ELISA kit (INOVA Diagnostics, San Diego, CA, USA) according to the manufacturer's instructions. ANA levels were determined according to fluorescence intensity on a scale of 0 (no fluorescence) to +3 (maximum brightness).

### Histological examination and scoring

Soft tissue biopsies of the heart, lungs, kidney, liver, pancreas, stomach, small intestine, colon, salivary glands, uterus and lacrimal glands were harvested into fresh Bouin's solution. Tissues were subsequently embedded in paraffin, and 2 *μ*m histological sections were stained with hematoxylin and eosin (H&E). Lymphocytic infiltrates into organs were scored from 0–3 (0=normal, 1=occasional small perivascular foci due to aging, 2=denser, well defined perivascular and peri-ductal foci, 3=extensive infiltration with parenchymal destruction). The kidneys were additionally assessed for evidence of glomerulonephritis (GN) and scored on a scale of 0–4 as previously described.^23^ All histological sections were examined with a compound microscope (Zeiss, Oberkochen, Germany), and images were photographed with a digital camera (AxioCam HRc, Zeiss).

### Cell culture and cell survival assays

CD4^+^CD8^+^, CD4^+^CD8^-^, CD4^-^CD8^+^ T-cells, B220^+^ B cells and Mac-1^+^Gr-1^hi^ granulocytes were sorted based on their surface marker expression profiles and cultured in 96-well flat bottom plates as previously described.^22^ Cells were then incubated in medium alone without cytokines (to mimic cytokine deprivation), or exposed to 1 *μ*M dexamethasone, 1 *μ*M etoposide, 240 nM ABT-737 (BH3 mimetic that inhibits BCL-2, BCL-XL and BCL-W), or FLAG-tagged Fas ligand (100 ng/ml; Enzo Life Sciences) cross-linked with anti-Flag M2 monoclonal antibody (2 *μ*g/ml; Sigma). At the indicated time points, cells were stained with 5 *μ*g/ml of propidium iodide (PI) along with FITC-conjugated annexin-V, and then subjected to flow cytometric analysis. Viable cells were determined as those negative for both PI and FITC-annexin-V (FITC^-^PI^-^).

### Statistical analyses

Animal survival curves were tested for statistical significance using the log-rank (Mantel-Cox) test. For comparison of mice weights, leukocyte numbers and serum ANA levels, the two-tailed Student's *t*-test was used (*P<0.05, **P<0.01, ***P<0.001 and ****P<0.0001).

## Abbreviations

ANA: anti-nuclear autoantibodies
APC: allophycocyanin
BCL-2: B cell lymphoma 2
BH: BCL-2 homology
CHO: Chinese hamster ovary
DKO: double knockout
DP: double positive
DN: double negative
FACS: fluorescence activated cell sorting
FITC: fluorescein isothiocyanate
FLCs: fetal liver cells
GN: glomerulonephritis
H&E: hematoxylin and eosin
MEF: mouse embryonic fibroblast
MOMP: mitochondrial outer membrane permeabilisation
PI: propidium iodide
R-PE: red phycoerythrin
SLE: systemic lupus erythematosus
TNFR: tumor necrosis factor receptor
TKO: triple knockout
WT: wild type
